# Molecular surveillance of shiga toxigenic *Escherichia coli* in selected beef abattoirs in Osun State Nigeria

**DOI:** 10.1038/s41598-021-93347-w

**Published:** 2021-07-07

**Authors:** Femi Ayoade, Judith Oguzie, Philomena Eromon, Omolola E. Omotosho, Tosin Ogunbiyi, Testimony Olumade, Kazeem Akano, Onikepe Folarin, Christian Happi

**Affiliations:** 1 Department of Biological Sciences, College of Natural Sciences, Redeemer’s University, PMB 230, Ede, Osun State Nigeria; 2 African Center of Excellence for the Genomics of Infectious Diseases (ACEGID), Redeemer’s University, PMB 230, Ede, Osun State Nigeria; 3 Biochemistry Unit, Department of Biological Sciences, Covenant University, Ota, Nigeria; 4 Department of Biological Sciences, Mountain Top University, KM 12, Lagos-Ibadan Expressway, Prayer City, Ogun State Nigeria

**Keywords:** Biochemistry, Biological techniques, Biotechnology, Microbiology, Molecular biology, Diseases

## Abstract

Shiga toxigenic strains of *E. coli* (STEC) known to be etiological agents for diarrhea were screened for their incidence/occurrence in selected abattoirs sources in Osogbo metropolis of Osun State, Nigeria using a randomized block design. Samples were plated directly on selective and differential media and *E. coli* isolates. Multiplex PCR analysis was used to screen for the presence of specific virulence factors. These were confirmed serologically as non-O157 STEC using latex agglutination serotyping kit. Sequence analysis of PCR products was performed on a representative isolate showing the highest combination of virulence genes using the 16S gene for identification purposes only. Results showed that the average cfu/cm^2^ was significantly lower in the samples collected at Sekona-2 slaughter slab compared with those collected at Al-maleek batch abattoir and Sekona-1 slaughter slab in ascending order at P = 0.03. Moreover, the average cfu/cm^2^ *E. coli* in samples collected from butchering knife was significantly lower when compared with that of the workers’ hand (P = 0.047) and slaughtering floor (P = 0.047) but not with the slaughter table (P = 0.98) and effluent water from the abattoir house (P = 0.39). These data suggest that the abattoir type may not be as important in the prevalence and spread of STEC as the hygiene practices of the workers. Sequence analysis of a representative isolate showed 100% coverage and 96.46% percentage identity with *Escherichia coli* O113:H21 (GenBank Accession number: CP031892.1) strain from Canada. This sequence was subsequently submitted to GenBank with accession number MW463885. From evolutionary analyses, the strain from Nigeria, sequenced in this study, is evolutionarily distant when compared with the publicly available sequences from Nigeria. Although no case of *E. coli* O157 was found within the study area, percent occurrence of non-O157 STEC as high as 46.3% at some of the sampled sites is worrisome and requires regulatory interventions in ensuring hygienic practices at the abattoirs within the study area.

## Introduction

Shiga toxigenic strains of *E. coli* (STEC) are widely recognized as major pathogens for public health problems in developing countries and represent the leading etiological agent of diarrhea^1,2^. Serogroup O157 has been the most reported STEC in humans and has been found to be responsible for most infections, sporadic cases and outbreaks of bacterial enteritis in humans, globally. On the other hand, there is increasing evidence that non-O157 STEC are acquiring greater importance as they are frequently associated with sporadic outbreak of both mild and severe STEC disease in humans globally^3,4^.

Many of the STEC strains are found in the gastrointestinal tracts of domesticated farm animals, hence these form the principal source of human infections. The most noxious *E. coli* strains are those that are able to produce putative accessory virulence factors such as intimin (encoded by eaeA) and the plasmid-encoded enterohemolysin, encoded by enterohemorrhagic *E. coli* (EHEC) hlyA. Moreover, *E. coli* strains producing Shiga toxin type 2 (*stx*_*2*_, encoded by *stx*_*2*_) appear to be more commonly responsible for serious complications such as HUS than those producing only Shiga toxin type 1^5^. On the other hand, *stx* production is not restricted to serogroup O157 strains, as over 100 STEC serotypes have been isolated from humans with diarrheal illness^4^.

Contaminated raw meat is one of the main sources of food-borne illnesses. The risk of the transmission of zoonotic infections is also associated with contaminated meat^6^. While meat is usually consumed well-done in Nigeria, thereby limiting infections from meat consumption, contamination of water bodies from abattoir waste constitutes significant environmental and public health hazards^6–8^. Bacteria from abattoir waste discharged into water columns can subsequently be absorbed to sediments, and when the bottom stream is disturbed, the sediment releases the bacteria back into the water columns presenting long-term health hazards^9^. In Nigeria, numerous abattoirs dispose of their effluents directly into the streams and waterways without any type of treatment and the butchered meat is washed by the same water^10^.

The incidence of Shiga toxigenic *E. coli* varies by country, where such data is available. Shiga toxigenic *E. coli* infections have been reported for most parts of the world, including a number of African countries^4^, however, specific incidence data are not always collected or readily available in most sub-saharan African countries, especially in Nigeria.

There are two main types of abattoirs available within the study area, namely, the slaughter slab and the batch systems. Slaughter slabs are the most commonly found in Nigeria. They are usually established and operated by municipal and local authorities. These operate in well-built areas and conform to a good extent with the WHO guidelines for abattoirs. On the other hand, the batch type of slaughter system are those where animals are killed and processed sometimes on bare floor or on corrugated roofing sheets placed on the floor. These are usually located in abandoned buildings or under the shade of trees and open exposed grounds that a butcher might find suitable for the business^11^.

The present surveillance work is aimed at screening for the incidence/occurrence of Shiga Toxigenic *Escherichia coli* (STEC) in selected abattoirs and retail meat sources in Osogbo metropolis in Osun State of Nigeria, using molecular and serotyping methods. The work is expected to positively inform on best practices in the local abattoirs, and enhance effective planning, implementation and evaluation of public health practice within the study area.

## Results

### Frequency of occurrence of *E. coli* contamination

**Figure 1:**
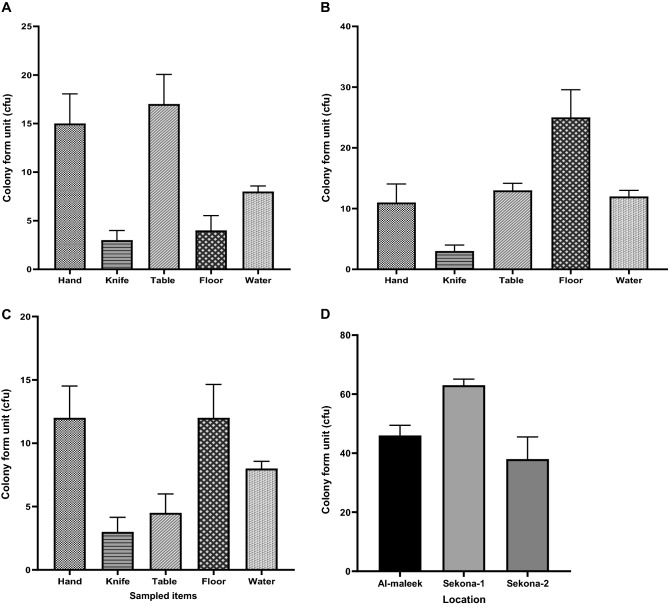
Average colony counts (cfu/ml) of *E. coli* colonies at the respective sampling locations (Workers’ hands, slaughtering knife, slaughtering tables, floor and effluent water) in Al-maleek (**A**), Sekona-1 (**B**), Sekona-2 and in all three sites (**D**) pooled together.

A total of 147 pure *E. coli* isolates were obtained from the entire study from the workers’ hands, butchering knives, slaughtering tables, floors and effluent water at the different abattoir locations. Based on observation and interaction with the abattoir workers, water for the washing of hands are usually provided in all the three abattoirs included in this study. Workers in any of the abattoir hardly used this for hand cleansing but preferred to wipe their hands with handkerchiefs instead. Al-maleek batch abattoir, overall mean colony forming units (cfu)/cm^2^ was 46 cfu/cm^2^ (SEM 3.5). Mean cfu/cm^2^ was significantly lowest in the samples taken from the butchering knife compared with workers’ hand, slaughter tables, slaughtering floors and effluent water [3 cfu/ml (SEM 1) versus 16 cfu/cm^2^ (SEM 3.1) versus 17 cfu/cm^2^ (SEM 3.1) versus 4 cfu/cm^2^ (SEM 1.5) versus 8 cfu/ml (SEM 0.6) respectively; P = 0.006] (Fig. 1A). In a post hoc comparison, the average cfu in samples from butchering knife and slaughtering floors were significantly lower than that of the workers’ hand (P = 0.04) and slaughtering table (P = 0.02).

At the Sekona-1 abattoir, overall mean colony forming units per cm^2^ of all samples was 63 cfu/cm^2^ (SEM 2.1). Mean cfu/cm^2^ was significantly higher in the samples taken from slaughtering floor compared with workers’ hand, butchering knife, slaughter tables, and effluent water from the abattoir house [25 cfu/cm^2^ (SEM 4.6) versus 11 cfu/cm^2^ (SEM 3.1) versus 3 cfu/cm^2^ (SEM 1) versus 13 cfu/cm^2^ (SEM 1.2) versus 12 cfu/ml (SEM 1) respectively; P = 0.007] (Fig. 1B). Multiple comparison showed, mean cfu/cm^2^ of cultures from all sampled items were similar except with the slaughtering floor.

At the Sekona-2 abattoir, overall mean cfu/cm^2^ was 38 cfu/cm^2^ (SEM 7.5). Mean cfu/cm^2^ was significantly lower in the culture of samples taken from butchering knife compared with those from workers’ hand, slaughter tables, slaughtering floors and effluent water [3 cfu/cm^2^ (SEM 1.2) versus 12 cfu/cm^2^ (SEM 2.5) versus 4.5 cfu/cm^2^ (SEM 1.5) versus 12 cfu/cm^2^ (SEM 2.6) versus 8 cfu/ml (SEM 0), respectively; P = 0.03] (Fig. 1C). In a post hoc analysis, mean colony forming units in samples from butchering knife was significantly lower compared with that of the workers’ hand (P = 0.047) and slaughtering floor (P = 0.047) but not with the slaughter table (P = 0.98) and effluent water from the abattoir house (P = 0.39).

When all data were pooled together, mean cfu/cm^2^ was significantly lower in the samples collected at Sekona-2 compared with those collected et al.-maleek and Sekona-1 [38 cfu/cm^2^ (SEM 7.5) versus 46 cfu/cm^2^ (SEM 3.5) versus 63 cfu/cm^2^ (SEM 2.1), respectively; P = 0.03] (Fig. 1D). In a post hoc analysis, mean colony form in samples collected from butchering knife was significantly lower compared with that of the workers’ hand (P = 0.047) and slaughtering floor (P = 0.047) but not with the slaughtering table (P = 0.98) and effluent water from the abattoir house (P = 0.39).

### Frequency of occurrence of STEC genes

**Figure 2:**
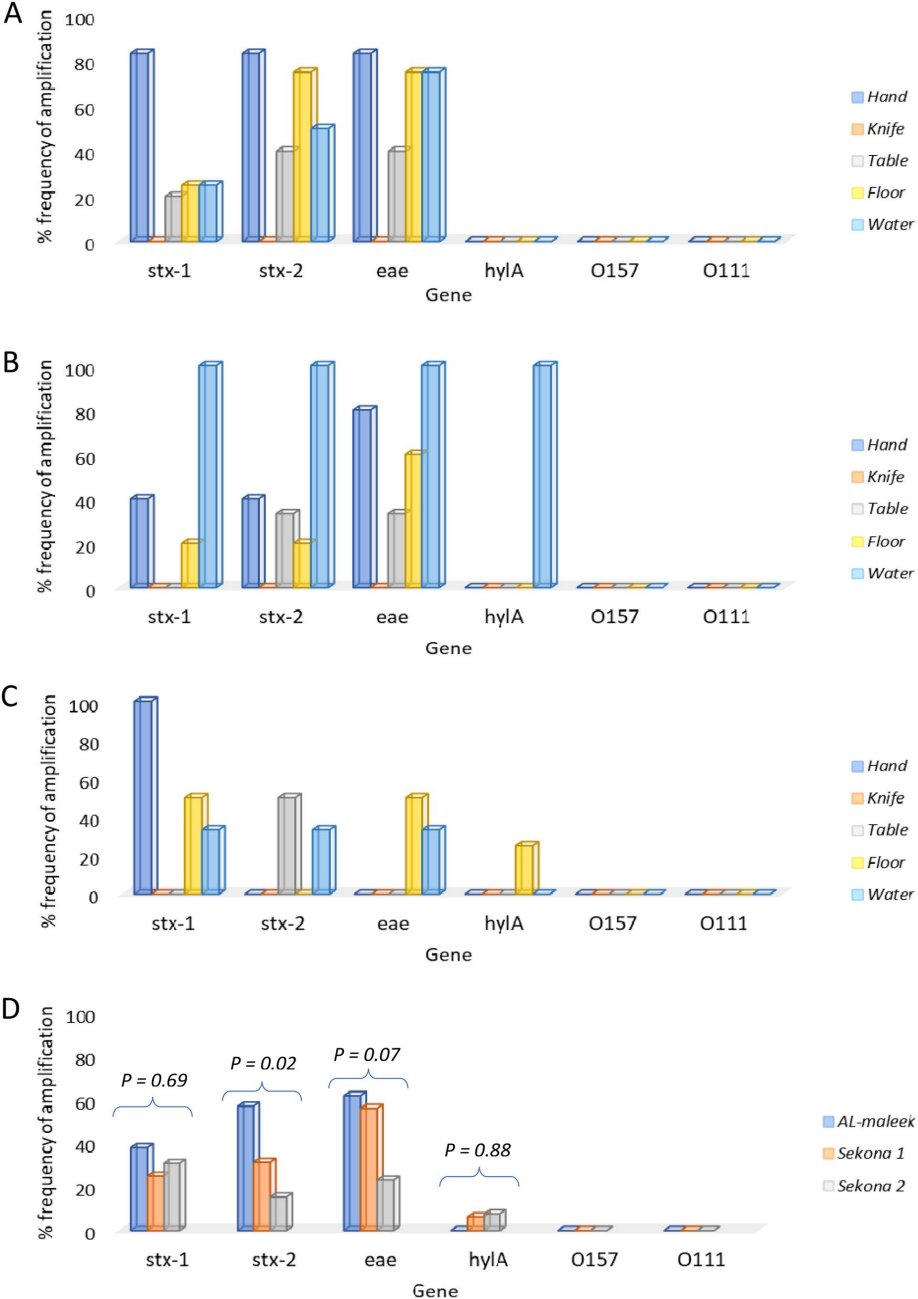
Distribution of stx-1, stx-2, eae, hylA, O157 and O111 genes among *E. coli* isolates from Al-maleek (**A**), Sekona-1 (**B**), Sekona-2 and in all three sites (**D**) pooled together.

A total number of pure isolates (147) were screened for the presence of six STEC genes. Of all the six genes screened, two (O157 and O111) did not amplify in any of the samples obtained from all three study sites (Fig. 2; Figure S1a–d, supplementary file). These results were corroborated through serotyping as none of the strains showed any visible agglutination with the O157 latex reagent. On the other hand, they caused a visible agglutination with the seroscreen latex reagent for detecting the 6 common non-O157STEC and were therefore identified as belonging to any of the non-O157 serogroups.

Similarly, *hylA* did not amplify in samples obtained from Al-maleek but amplified in samples from Sekona-1 and from Sekona-2 (Fig. 2D).

When all data from all sampled items were pooled together according to sample sources, occurrence of *stx*_*2*_ gene was significantly more common in Al-maleek compared with Sekona-1 and Sekona-2. [12 of 21 isolates (i.e. 57.1%) versus 5 of 16 (i.e. 31.3%) versus 2 of 13 (i.e. 15.4%) in Al-maleek, Sekona-1 and Sekona-2, respectively; P = 0.02] (Fig. 1D). However, distribution of *stx*_*1*_ and *eae* genes were similar in the isolates from all the three study locations. For *stx*_*1*_: [8 of 21 isolates (i.e. 38.1%) versus 4 of 16 (i.e. 25%) versus 4 of 13 (i.e. 30%) in Al-maleek, Sekona-1 and Sekona-2, respectively; P = 0.69] (Fig. 1D). For *eae*: [13 of 21 (i.e. 61.9%) versus 9 of 16 (i.e. 56.1%) versus 3 of 13 (i.e. 23.1%) in Al-maleek, Sekona-1 and Sekona-2, respectively; P = 0.07] (Fig. 2D). In further analysis, *eae* was significantly more common in Al-maleek compared with Sekona-2 (P = 0.04) but not with Sekona-1 (P = 0.99) (Fig. 2D).

It is noteworthy that one isolate (Se1-5-W5) from effluent water samples taken from Sekona-1 abattoir amplified 4 out of the 6 targeted genes (*hly*A, *stx*_*1*_, *stx*_*2*_ and *eae*). This isolate was singled out and set aside for molecular identification.

### Sequencing and BLAST analysis of representative sample

**Figure 3:**
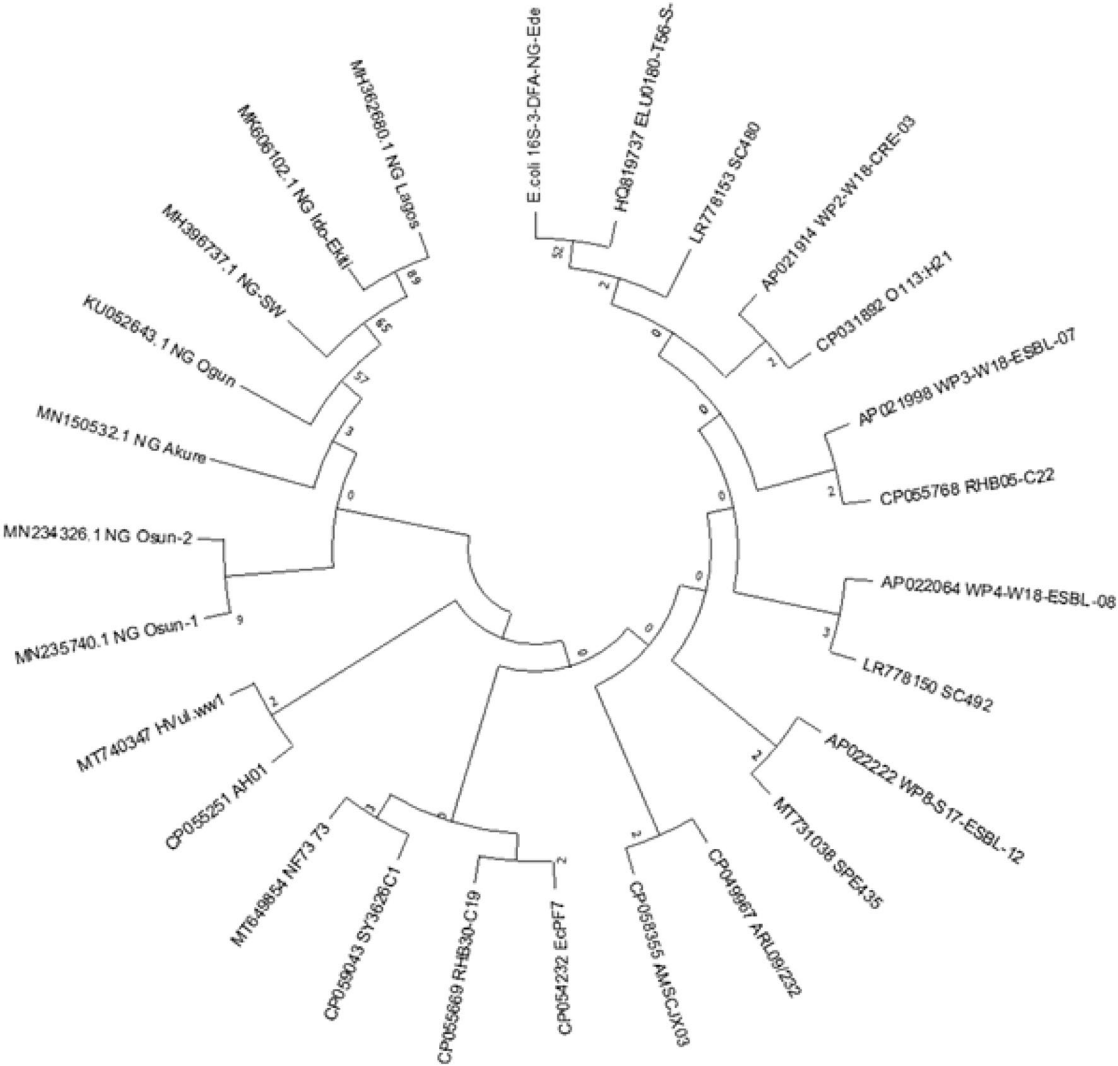
Phylogenetic analysis of *E. coli* sequence from Beef abattoir compared with publicly available sequences. Evolutionary analyses and phylogenetic tree were conducted in MEGA X version 7 (https://www.megasoftware.net/).

The only isolate in which 4 out of the total of 6 genes considered were amplified was the isolate with identification number Se1-5-W5 (Fig. 2D). This isolate which was obtained from the effluent water from the Sekona abattoir was sequenced, primarily for identification by targeting the 16S gene and compared with publicly available sequences available on NCBI for phylogenetic analysis (Fig. 3). Following BLAST analysis, the sequence showed 100% coverage with 97% percentage identities to the ribosomal RNA gene of two uncultured organism clones with GenBank Accession numbers HQ791150.1 and HQ819737.1, a strain of Shigella spp. from London, UK, including a strain of *Escherichia coli* O113:H21 (GenBank Accession number: CP031892.1) strain from Canada with 96 percentage identity. From analyses, the strain from Nigeria, sequenced in this study, is evolutionarily distant when compared with the publicly available sequences from Nigeria (Fig. 3). The isolated 16S rRNA sequence was subsequently submitted to GenBank and registered with accession number MW463885.

## Discussion

The present study investigated the prevalence of Shiga-toxigenic *E. coli* from selected beef abattoirs in Osun State. The results revealed that the average colony count (cfu/cm^2^) of distinct isolates of *E. coli* was significantly lower in the samples collected at Sekona-2 slaughter slab when compared with those collected at Almaleek (a batch type abattoir) and Sekona-1 slaughter slab type abattoir in ascending order (Fig. 1D). The current study shows the prevalence of *E. coli* was similar at the Sekona-1 and Al-maleek abattoirs. This observation contradicts previous reports that favor Slaughter slabs over batch type simply because slaughter slabs operate in structurally well-built areas as opposed to batch type abattoirs where animals are killed and processed typically on corrugated roofing sheets placed on the floor^11^. This may indicate that mere structural design of an abattoir may not be a sole yardstick for rating propensity for *E. coli* contamination, evaluation of the abattoir practice and environmental burden must be considered.

In this evaluation, it was well demonstrated that hygienic practices of the abattoir workers is a more important factor in determining the rate of *E. coli* contamination in the abattoir setting than the structure of the abattoir, that is, whether it is batch or slaughter slab type. For instance, the post hoc analysis of the prevalence of *E. coli* colonies recovered from the sampled areas showed that the average cfu/cm^2^ was consistently significantly higher on the workers’ hand as well as on the slaughtering floor when compared with the colony count on the butchering knife (Fig. 1D). It appears that contamination is more likely to originate from the human activity during meat processing and that human activities may facilitate spreading the contaminants to the tables and floors and then to the effluent water.

Furthermore, the frequency of occurrence of the STEC genes data revealed that only four of the six targeted genes amplified in the *E. coli* colonies, the four genes include *stx*_*1*_, *stx*_*2*_, *eae* and *hylA*. The non-occurrence of *hlyA* in samples obtained from Al-maleek batch abattoir further suggest that hygiene practices of the abattoir workers is more important in determining the rate of contamination rather than the abattoir type, since slaughter slabs are supposed to be structurally superior to batch type abattoirs. This hypothesis is further confirmed by the data showing that the workers’ hands consistently displayed higher frequency of amplification of STEC genes when compared with other sample sources (Fig. 2A–C).

In addition, the present results agree with previous reports showing that cattle are the primary source of Shiga-toxigenic strains of *E. coli*^12–14^. Our results are consistent with reports from similar studies where prevalence rates of up to 67% of STEC have been reported in cow hides, a human delicacy in the study area^15^. Similarly, several authors have reported high prevalence rates of STEC and non-STEC in meat sampled from Nigerian abattoirs^16,17^. The selected abattoirs in the present study primarily process cow meat.

Moreover, results from the present study showing that none of the isolates tested positive for the presence of O157 and O111 genes is not surprising. In studies where these have been reported in south-western Nigeria, the incidence rates has been found to be very low. For example, in nearby Ado-Ekiti^18^, a prevalence rate of 4.1% was reported in healthy cattle which is considerably lower than 14% non-O157 STEC strains recovered from cattle feces in Alberta, Canada^19^. The present study adds to the body of evidence that ruminants, particularly cattle represent the most important reservoir and source of human infection as a result of exposure to animal manure by contamination of food and water.

In addition, the present study shows the absence of O157 STEC strains within the study area (Figs. 1 and 2; Supplementary Figure S1), this is in contrast to reports from Lagos (which falls within the south-western geographical zone of Nigeria) where the isolation of *E. coli* O157:H7 from some food animals including goats have been reported^20^. On the other hand, the presence of non-O157 STEC strains at most of the study sites sampled in this study remains worrisome and a source for concern from the stance of public health.

Isolate Se1-5-W5 recovered from water samples from the Sekona-1 abattoir amplified 4 out of the 6 STEC genes screened for in the present study, representing the highest number and diversity of amplified genes found in the total of 50 isolates assessed. For this reason, this isolate was sequenced for identification purposes only and was identified as O113:H21 serotype. This particular strain has however been observed to amplify the *eae* gene (Fig. 2). Although not commonly encountered, *eae*-positive O113:H21 have been reported severally in literature^21–23^, with most of the reports coming from Brazil, where this serotype is the most frequent STEC serotype isolated from cattle and sometimes in humans^24^. The present result calls for further studies in the areas of sequencing for STEC genes in the selected isolates and assessment of the prevalence of O113:H21 serotypes in the Nigerian context.

On the other hand, even though the analysis of the sequencing data from the present study suggest closeness to a strain of *Escherichia coli* O113:H21 (GenBank Accession number: CP031892.1) strain from Canada with 96 percentage identity, this is distant from the definition of a species “match” which typically should be less than 1% divergence, that is, greater than 99% similarity. The data from the present study with 96% similarity may not be sufficient to comment on identification but only relatedness, since the only other organisms with closer relatedness (97%) are from uncultured organism clones from New York, USA and a *Shigella* strain from London, U.K.

Results from the present study records no case of *E. coli* O157 within the study area, however, percentage occurrence of non-O157 STEC was as high as 46.3% at some of the sampled sites (Figs. 1 and 2). Up until now, zoonotic *E. coli* transmission was assumed to be more problematic in developed nations with highly industrialized agricultural systems, however, our results show that this may not be the case and the lack of reporting of high numbers of occurrence of *E.coli* transmissions may be due to lack of a surveillance system. Our study underscores the importance of developing a Laboratory-based Enteric Disease Surveillance (LEDS) system in Nigeria to stem the spread of noxious enteric diseases.

## Materials and methods

### Sample collection

Samples were obtained from 3 different sites of abattoirs within 50 km radius of Oshogbo, the capital city of Osun state Nigeria. Oshogbo is located at coordinates: 7°46′N 4°34′E and has a total area size of 47 km^2^ (18 sq. miles). The 3 abattoirs were selected for being the busiest abattoirs primarily devoted to slaughtering cattle within the study area, processing at least ten but not more than fifty cows slaughtered daily. One of the selected abattoirs, Al-maleek is a batch type while the other 2 abattoirs, Sekona-1 and Sekona-2 are slaughter slab type abattoirs. Using sterile swab (3 cm long and 1 cm diameter) held by wooden stick and moistened with 0.1% peptone water, samples were obtained from slaughtering floors, slaughtering tables, butchering knives and worker’s hands. Briefly, an area of 25 cm^2^ (5 cm × 5 cm) was marked on the surface from which sample is to be taken, the swabs were rubbed on the sites for around 30 s and then transferred to individual screw-capped test tube containing sterile maintenance medium (0.85% NaCl and 0.1% peptone). Composite effluent water samples were collected using the grab method in sterile screw cap tubes. Samples were taken in triplicates and transported immediately to the laboratory for analyses. Samples were enumerated by using appropriate sterile dilution and spread plate methods, samples were incubated at 30 °C for 18–24 h. The distinct colonies on the plates were counted using a colony counter and the mean bacteria count per ml was calculated using the formula by dividing the average value of colony forming units on enumerated plates by the volume of sample dispensed unto agar and multiplying the product by the dilution factor. The average number of colonies in a particular dilution was expressed as the number of organisms or colony forming unit (CFU) of each sample and calculated into its log value. The microbiological data were expressed in log cfu/cm^2^ and log cfu/ml in case of swab and water respectively. A randomized block design was used. This design made it possible to compare the expected differences between frequency of occurrence of *E. coli* and STEC genes data from the two abattoir types (the blocks), while samples were collected from the slaughtering floors, slaughtering tables, butchering knives and worker’s hands and effluent water samples (the treatments).

### Consent

The purpose of the study was explained to the abattoir workers at each location with translation provided in the local language (Yoruba) and informed consent was obtained before taking sterile swabs of the workers’ hands and knives. Each participant’s right to refuse taking swabs from their knives or hands was respected at all times. This research was conducted according to the approved guidelines set by the Redeemer’s University Research Ethics Committee, Redeemer’s University, Ede, Osun State, Nigeria. This research was approved by the Redeemer’s University Research Ethics Committee, Redeemer’s University, Ede, Osun State, Nigeria.

### Identification and serotyping of *E. coli* isolates

The samples were screened for the presence of members of the family Enterobacteriaceae, specifically *E. coli*, and these were targeted for use as indicators of microbial contamination. The inocula were plated directly on selective and differential media, namely, MacConkey (MAC) and Eosin Methylene Blue (EMB) agar. Furthermore, gas production and indole tests when isolates were grown at 44 °C were used in order to confirm the isolates as *E. coli*. Subsequently, the confirmed *E. coli* isolates were stocked at – 4 °C and until used for downstream analyses such as genomic DNA extraction. The strains of *E. coli* O157 were identified culturally on the basis of inability to ferment sorbitol on SMAC agar and confirmed serologically as O157 by latex agglutination serotyping kit (Dryspot *E. coli* O157 latex test) for *E. coli* O157 (Oxoid, Basingstoke, UK) and (Dryspot *E. coli* serocheck and seroscreen latex test) for the detection of six non-O157 serogroups O26, O91, O103, O111, O128 and O145. The kit was used according to the manufacturer’s specifications. The test reaction comprised of blue latex particles sensitized with specific rabbit antibody reactive with the relevant serogroup of *E. coli* (e.g., O157, O26, O111 etc.) as indicated on test card.

### Statistical analysis

Data were analyzed using version 7 of Epi-Info software26 and GraphPad Prism version 8^25,26^. Simple graphs were plotted using GraphPad Prism version 8 and Microsoft Excel version 16^27^. Proportions were compared by calculating χ^2^ using Yates’ correction, Fisher’s exact or Mantel Haenszel tests. Normally distributed, continuous variables were compared by analysis of variance (ANOVA). Post-hoc comparisons of multiple parameters, where necessary, were done using Tukey Honestly Significant Difference Test (Tukey HSD). p values of < 0.05 were taken to indicate significant differences.

### DNA extraction, PCR amplification and fragment purification

Genomic DNA was extracted from 50 randomly selected isolates from the entire study using the Excel Random Number Generator 28. Specifically, stock cultures of *E. coli* were incubated overnight in tryptic soy broth (TSB; Oxoid, England, U.K) then sub-cultured in fresh TSB (20 × dilution) and incubated for approximately 1 h. The sub-culture was grown to reach ca. 4 × 108 CFU ml^−1^, with an optical density between 0.5 and 0.6 at 600 nm (path length 1 cm). All broth cultures were grown at 37 °C and aerated with orbital shaking at 200 rpm, 200 μl of this was used for DNA extraction. Quick DNA™Fungal/Bacterial Miniprep kit was used in extracting the DNA by following manufacturer’s instructions. The multiplexed PCR reactions were sectioned into 2 assays, namely assay 1 (comprising 4 sets of primers) and assay 2 (comprising 2 sets of primers) (Table 1). An aliquot of 5 μl of the extracted DNA was used as a template in a 25 μl PCR reaction mixture containing illustra™ PuReTaq™ Ready-To-Go™ PCR Beads, 1 μl each of forward and reverse for each of the primers, including 1.5 mM MgCl_2_ and 16.5 μl of double distilled water. Samples were subjected to 35 PCR cycles, each consisting of 1 min of denaturation at 95 °C; 2 min of annealing at 65 °C for the first 10 cycles, decrementing to 60 °C by cycle 15; and 1.5 min of elongation at 72 °C, incrementing to 2.5 min from cycles 25 to 35. PCR reaction mixtures were electrophoresed on 2% agarose gels and stained with ethidium bromide^28^.

**Table 1:** The PCR primers.

Primer	Sequence (5′–3′)	Specificity^1^	Amplicon size (bp)
**Assay 1**			
stx1F	ATAAATCGCCATTCGTTGACTAC	nt 454–633 of A subunit coding region of *stx*	180
stx1R	AGAACGCCCACTGAGATCATC		
stx2F	GGCACTGTCTGAAACTGCTCC	nt 603–857 of A subunit coding region of *stx*2 (including *stx*2 variants)	255
stx2R	TCGCCAGTTATCTGACATTCTG		
eaeAF	GACCCGGCACAAGCATAAGC	nt 27–410 of *eaeA* (this region is conserved between EPEC and STEC)	384
eaeAR	CCACCTGCAGCAACAAGAGG		
hlyAF	GCATCATCAAGCGTACGTTCC	nt 70–603 of EHEC *hlyA*	534
hlyAR	AATGAGCCAAGCTGGTTAAGCT		
**Assay 2**			
O157F	CGGACATCCATGTGATATGG	nt 393–651 of *rfbE*O157:H7	259
O157R	TTGCCTATGTACAGCTAATCC		
O111F	TAGAGAAATTATCAAGTTAGTTCC	nt 24–429 of ORF 3.4 of *E. coli* O111 *rfb* region	406
O111R	ATAGTTATGAACATCTTGTTTAGC		

^1^*nt* nucleotide, *ORF* open reading frame^29^.

### Analysis of *E. coli* cultures by multiplex PCR and sequencing of representative isolates

Cultures from the *E. coli* isolates obtained from the sample locations were analyzed by PCR as described earlier, the O157 and O111 genes did not amplify in both the control and test isolates. However, during the course of data analyses, a particular isolate with isolate identification number Se1-5-W5, obtained from water effluent from the Sekona-1 abattoir site, observed to express the highest number of targeted genes was singled out for the purpose of identification only. Subsequently, Sanger sequencing was applied to this particular representative strain that displayed the highest number and diversity of targeted STEC genes for identification purposes only.

25 ng of the PCR product from the amplification of the 16S gene of the representative strain was sequenced using the BigDye Terminator v3.1 on an ABI 3500 Genetic Analyzer from Life Technologies. The raw reads came from this cycle sequencing procedure. The whole genome was not sequenced.

Raw reads from Sanger sequencing were trimmed using BioEdit Sequence Alignment Editor (version 7.2.6) (https://bioedit.software.informer.com) with manual base calling where necessary. The resulting consensus sequence was then subjected to BLAST (Basic Local Alignment Search Tool) analysis for the identification of the organism. Alignment of the sequence, with other publicly available sequences from GenBank, was carried out using the ClustalW algorithm in Geneious Prime (version 2020.2.4). Evolutionary analyses were conducted in MEGA X^29^.

The evolutionary history was inferred by using the Maximum Likelihood method and Hasegawa-Kishino-Yano model^30^. The tree with the highest log likelihood (− 3908.74) is shown. The percentage of trees in which the associated taxa clustered together is shown next to the branches. Initial tree(s) for the heuristic search were obtained automatically by applying Neighbor-Join and BioNJ algorithms to a matrix of pairwise distances estimated using the Maximum Composite Likelihood (MCL) approach, and then selecting the topology with superior log likelihood value. This analysis involved 25 nucleotide sequences from the 16S sequences publicly available on the NCBI database used for the phylogenetic analyses. Evolutionary analyses were conducted in MEGA X ^29^.

## Supplementary Information

Supplementary Figure S1.
