# Supplementary material for: Molecular surveillance of shiga toxigenic Escherichia coli in selected beef abattoirs in Osun State Nigeria
Source: Sci Rep. 2021 Jul 7;11:13966. doi: 10.1038/s41598-021-93347-w (PMC8263744; doi:10.1038/s41598-021-93347-w)
Supplement: Supplementary file 1 — Supplementary Figure S1. [file 41598_2021_93347_MOESM1_ESM.docx]

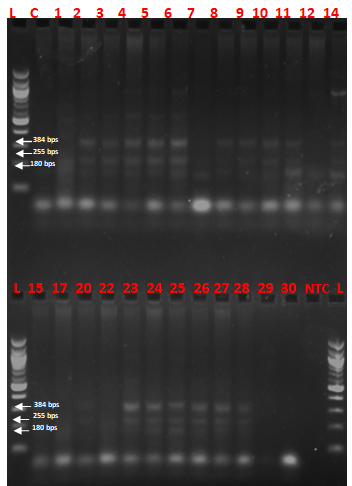


Figure 3a (Supplementary) : Characterization of *E coli* strains by multiplex PCR assay strains were classified based on the visible banding patterns expected mobilities for the various specific PCR products (see Table 1).


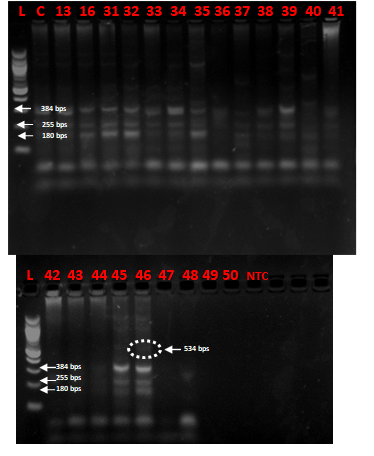


Figure 3b (Supplementary): Characterization of *E coli* strains by multiplex PCR assay strains were classified based on the visible banding patterns expected mobilities for the various specific PCR products (see Table 1). The lone sample with faint band showing at 534 bps was selected for sequencing, see sequencing result on Figure 4.


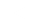

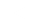

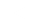


**180 bps**

**255 bps**

**384 bps**

**384 bps**

**255 bps**

**180 bps**

**534 bps**


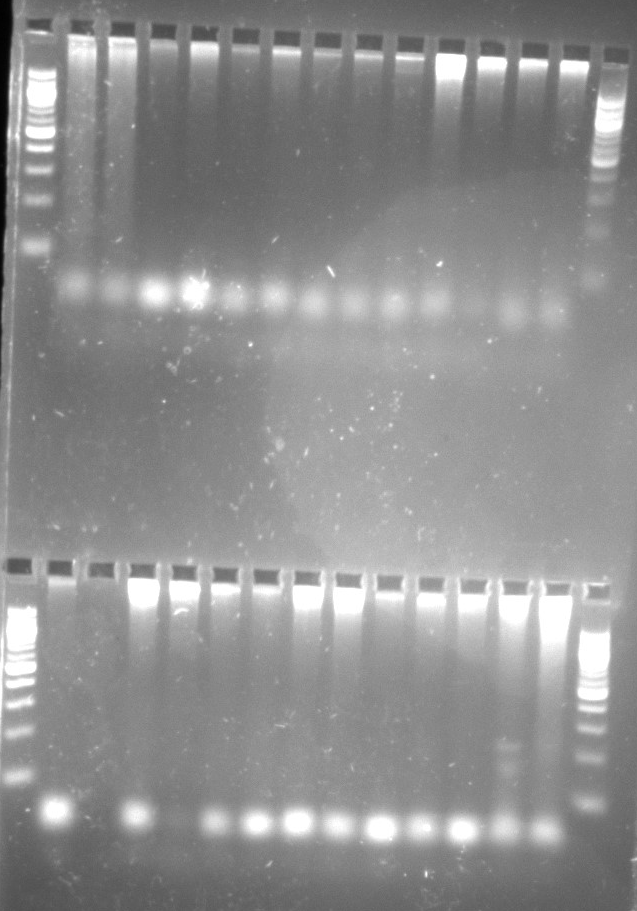


**L 14 15 16 17 18 19 20 21 22 23 24 25 26**

Figure 3c (Supplementary): Characterization of *E coli* strains by multiplex PCR assay (2) strains were classified based on the visible banding patterns expected mobilities for the various specific PCR products (see Table 1). No bands were observed in the expected band sizes.

**L 1 2 3 4 5 6 7 8 9 10 11 1213**

**L 14 15 16 17 18 19 20 21 22 23 24 25 26**


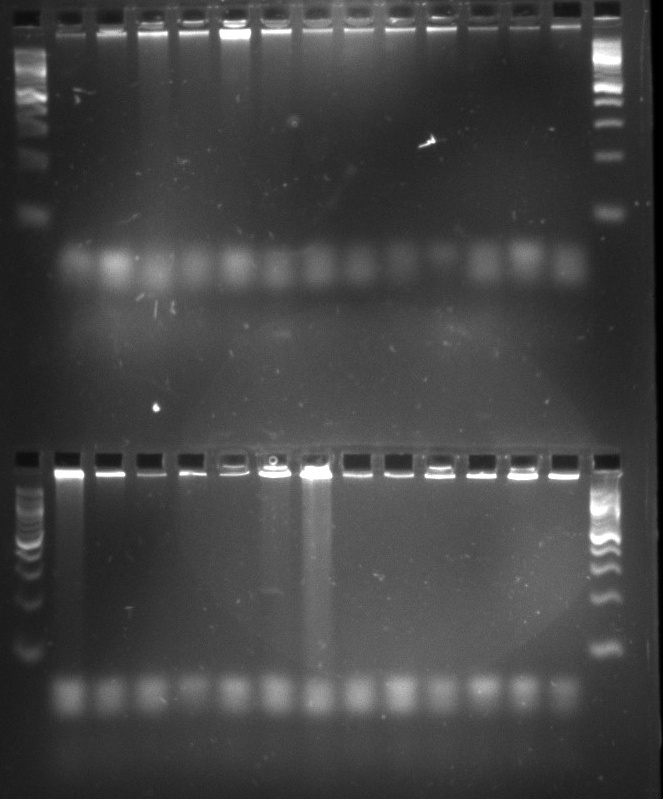


**L 27 28 29 30 31 32 33 34 35 36 37 38 39 L**

**L 40 41 42 43 44 45 46 47 48 49 50 L**

Figure 3d (Supplementary): Characterization of E coli strains by multiplex PCR assay (2) strains were classified based on the visible banding patterns expected mobilities for the various specific PCR products (see Table 1). No bands were observed in the expected band sizes.

Legend: The lane numbers are matched with the corresponding isolate Identification numbers and the sample sources as follows, 1-Alh-A3-3 (Al-maleek, hands); 2- Alh-B3-5 (Al-maleek, hands); 3- Alh-A3-5 (Al-maleek, hands); 4- Alh-C3-1 (Al-maleek, hands); 5- Alh-B5-4 (Al-maleek, hands); 6- Alh-A3-4 (Al-maleek, hands); 7- Se2h-C1-1 (Sekona-2, hands); 8- Se1h-A5-1 (Sekona-1, hands); 9- Se1h-C3-4 (Sekona-1, hands); 10- Se1h-A1-4 (Sekona-1, hands); 11- Se1h-A1-4 (Sekona-1, hands); 12- Se1h-B3-5 (Sekona-1, hands); 13- Se1FA3-4 (sekona-1, floor); 14- Se2FC1-2 (Sekona-2, floor); 15- Se2FC1-5 (Sekona-2, floor); 16- Se1F3-1 (Sekona-1, floor); 17- Se2FA3-3 (Sekona-2, floor); 18- Se1FC1-3 (Sekona-1, floor); 19- Se1FA3-1 (Sekona-1, floor); 20- Se2FC5-3 (Sekona-2, floor); 21- Se1FC1-3 (Sekona-1, floor); 22- AlFA-3 (Al-maleek, floor); 23- AlfC5-3 (Al-maleek, floor); 24- AlFC3-4 (Al-maleek, floor); 25- AlF-3-2 (Al-maleek, floor); 26- Al-3-W3 (Al-maleek, water); 27- Al-3-W4 (Al-maleek, water); 28- Al-1-W2 (Al-maleek, water); 29- Se2-3-W3 (Sekona-2, water); 30- Se2-5-W1 (Sekona-2, water); 31- Se2-3W1 (Sekona-2, water); 32- Se1kA-3-2 (Sekona-1, knife); 33- ALTB-1 (Al-maleek, table); 34- AlTC-5 (Al-maleek, table); 35- AlTA-5 (Al-maleek, table); 36- AlTC-1 (Al-maleek, table); 37- ALTB-5 (Al-maleek, table); 38- Se2TC-3-4 (Sekona-2, table); 39- Se1TC-3-3 (Sekona-1, table); 40- Se2-TB-1-4 (Sekona-2, table); 41- Se1TB-3-1 (Sekona-1, table); 42- Se1TA-3-2 (Sekona-1, table); 43- Alk-B3-1 (Al-maleek, knife); 44- AlkB3-2 (Al-maleek, knife); 45- Se2K-C3-2 (Sekona-2, knife); 46- Se1-5-W5 (Sekona-1, water); 47- Se2K-BC-2 (Sekona-2, knife); 48- Se1KB5-5 (Sekona-1, knife); 49- Se2K-A3-3 (Sekona-2, knife); 50- Al-1-W1 (Al-maleek, water).
